# Tuberculose péritonéale pseudo tumorale mimant un cancer ovarien: un diagnostic différentiel important à considérer

**DOI:** 10.11604/pamj.2016.25.193.10929

**Published:** 2016-11-25

**Authors:** Mounir Moukit, Fatimazahra Ait El Fadel, Jaouad Kouach, Abdellah Babahabib, Mohammed Dehayni, Driss Moussaoui Rahali

**Affiliations:** 1 Service de Gynécologie et Obstétrique, Hôpital Militaire d’Instruction Mohammed V, Rabat, Maroc; 2 Faculté de Médecine et de Pharmacie, Université Mohammed V, Rabat, Maroc; 3 Pôle de Chirurgie Viscérale et de Gynécologie-Obstétrique

**Keywords:** Tuberculose péritonéale, cancer ovarien, laparotomie, Peritoneal tuberculosis, ovarian cancer, laparotomy

## Abstract

La tuberculose est une maladie infectieuse curable qui peut simuler dans sa localisation péritonéale un cancer ovarien avancé conduisant ainsi à une chirurgie étendue et inutile souvent chez des femmes en âge de reproduction. Nous rapportons un nouveau cas de tuberculose péritonéale pseudo tumorale chez une patiente âgée de 43 ans chez qui le diagnostic d’un cancer ovarien avec carcinose péritonéale avait été suspecté. La laparotomie exploratrice avec examen histologique extemporané ont permis de confirmer le diagnostic de tuberculose péritonéale. La patiente a bien répondu au traitement antituberculeux selon le protocole 2ERHZ/4RH.

## Abstract (en)

Peritoneal tuberculosis is a curable infectious disease that can simulate advanced ovarian cancer, leading to extensive and unnecessary surgical procedures commonly performed on women of reproductive age. We report a new case of pseudo-tumoral peritoneal tuberculosis in a 43-year old female patient with suspected ovarian cancer associated with peritoneal carcinomatosis. The diagnosis of peritoneal tuberculosis was based on exploratory laparotomy with extemporaneous histological examination. The patient responded well to anti-tuberculosis treatment according to 2ERHZ/4RH protocol.

## Introduction

Le cancer de l’ovaire est le plus difficile des cancers gynécologiques à diagnostiquer à cause de ses symptômes peu spécifiques. La tuberculose péritonéale représente l’un des diagnostics différentiels, car elle peut mimer un tableau de cancer ovarien évolué tout en augmentant les marqueurs sériques classiquement associés à ce cancer. Dans une étude menée par Oge et al, parmi les 612 patientes opérées pour suspicion de cancer ovarien, 20 cas (soit 3.2%) étaient une tuberculose péritonéale confirmé en postopératoire [1]. Nous rapportons un nouveau cas de tuberculose péritonéale simulant un cancer ovarien évolué et nous discutons, à travers une revue de littérature, les difficultés diagnostiques et thérapeutiques imposées par cette entité.

## Patient et observation

**Figure 1:**
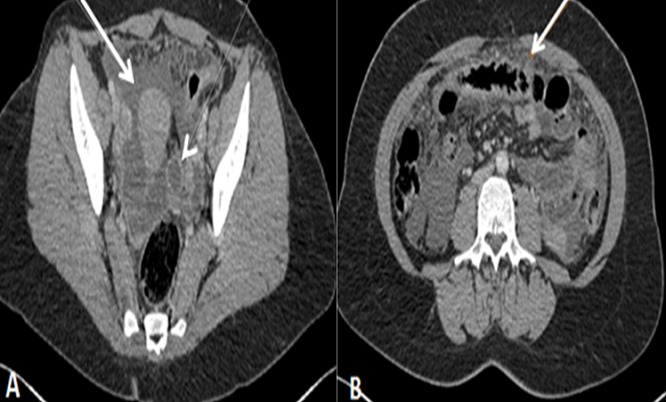
Coupes tomodensitométriques de l’étage abdominopelvien. A: aspect hétérogène de l’ovaire gauche (tête de la flèche) mesurant 45x40 mm, associé à un épanchement péritonéal (flèche blanche). B: épaississement mésentérique (flèche blanche)

**Figure 2:**
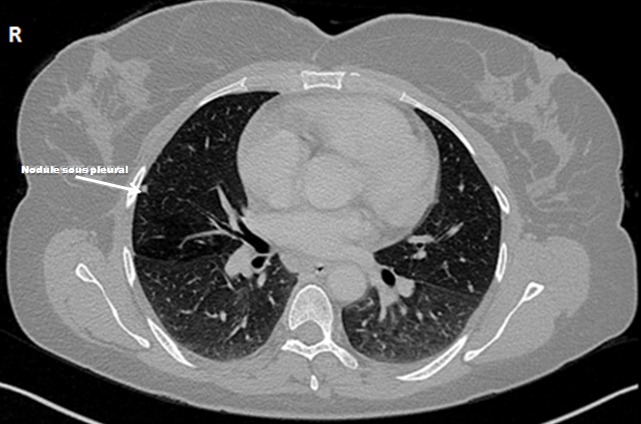
Lésions nodulaires sous pleurales intéressant le lobe moyen et inférieur droit sur une coupe tomodensitométrique

**Figure 3:**
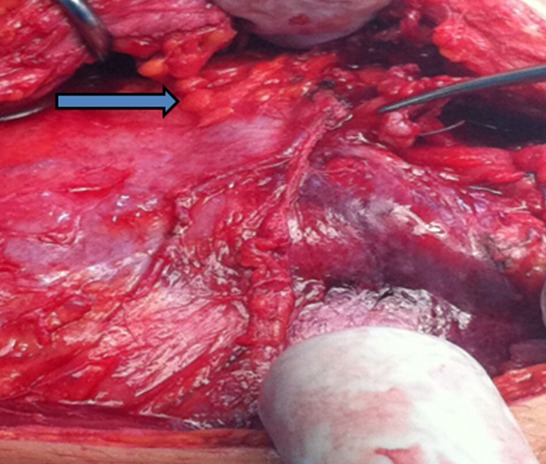
Vue per opératoire objectivant une inflammation péritonéale diffuse associée à des multiples formations blanchâtres au niveau du péritoine et l’épiploon (flèche bleue)

**Figure 4:**
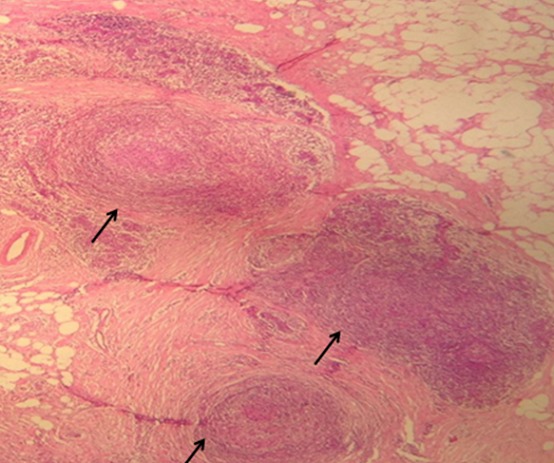
Aspect microscopique du granulome épithèloïde et gigantocellulaire avec nécrose caséeuse (flèches noires)

Une patiente âgée de 43 ans, d’origine rural, multipare, sans antécédents pathologiques notables, admise dans notre formation pour des douleurs abdomino-pelviennes chroniques, évoluant dans un contexte d’anorexie et d’amaigrissement non chiffré. L’examen clinique à l’admission objectivait un abdomen légèrement distendu avec matité des flancs. L’échographie abdomino-pelvienne montrait un aspect hétérogène de l’ovaire gauche, mesurant 5cm de grand axe, baignant dans un épanchement péritonéal de moyenne abondance. L’utérus était de taille normale, d’échostructure homogène avec un endomètre fin. L’ovaire controlatéral était d’aspect normal. Le complément tomodensitométrique confirmait l’aspect hétérogène de l’ovaire gauche et l’épanchement péritonéal, associé à un épaississement mésentérique (Figure 1). A l’étage thoracique, on notait la présence des lésions nodulaires sous pleurales intéressant le lobe moyen et inférieur droit (Figure 2). Le CA125 était augmenté à 445,2 UI/ml alors que les autres marqueurs tumoraux (antigène carcino-embryonnaire, alfa fœtoprotein et l’HCG) étaient normaux. Le reste du bilan biologique était normal. Devant ce tableau, le diagnostic d’un cancer ovarien gauche avec ascite et carcinose péritonéale était évoqué sans exclure une tuberculose péritonéale secondaire à une localisation pulmonaire séquellaire. Une laparotomie médiane exploratrice a était réalisée mettant en évidence la présence d’une inflammation péritonéale diffuse associée et de multiples formations blanchâtres au niveau du péritoine et l’épiploon (Figure 3). Le pelvis était blindé et adhérentiel gênant l’exploration de l’utérus et des annexes. Une biopsie des granulations blanchâtres était réalisée. L’examen anatomo-pathologique extemporané objectivait la présence d’un granulome épithèloïde et gigantocellulaire avec nécrose caséeuse en faveur d’une tuberculose péritonéale (Figure 4). L’évolution était bonne sous traitement antibacillaire selon le protocole 2ERHZ/4RH, avec un recul de 18 mois.

## Discussion

La localisation péritonéale pseudo tumorale de la tuberculose est une forme clinique rare, avec une fréquence estimée de 1 à 3% selon les séries [1, 2]. Cette fréquence est plus importante et pourrait doubler, voire tripler chez les sujets séropositifs. Elle peut toucher toutes les tranches d’âge avec une prédilection chez les femmes entre 20 et 50 ans [1]. La greffe péritonéale du Mycobacterium Tuberculosis se fait par voie hématogène; principalement à partir d’une primo infection pulmonaire passer souvent inaperçue, comme dans le cas de notre patiente, plus rarement après une primo infection gastro-intestinale [3]. L´absence de vaccination est le principal élément incriminée dans l’atteinte péritonéale à côté des autres facteurs tels que le manque d´hygiène et la précarité des conditions socio-économiques. Notre patiente avait tous ces facteurs de risque. Cliniquement, la tuberculose péritonéale peut mimer un tableau de cancer ovarien avancé. En effet, les douleurs pelviennes, la distension abdominale, l´amaigrissement et la palpation d’une masse abdominopelvienne peuvent être présent dans les deux pathologies. Cependant, la recherche d´autres signes à savoir les troubles menstruels (55% des cas dans la série d’Oge et al), digestifs et urinaire peut orienter le diagnostic [1]. L’infertilité est révélatrice dans 5 à 10% des cas [4]. Une association avec d´autres localisations notamment pulmonaire est à rechercher, mais leur absence n’élimine pas le diagnostic [1]. Les techniques d’imagerie comme l’échographie et la tomodensitométrie peuvent parfois orienter le diagnostic. En effet, l’existence d’une ascite avec des septas, l’épaississement péritonéal, son rehaussement hétérogène, l’existence de foyers hypodenses en rapport avec la nécrose caséeuse et les adénopathies suggèrent l’origine tuberculeuse, de même que l’existence des séquelles pleuro-parenchymateuses de tuberculose pulmonaire (cas de notre patiente), moins souvent des lésions évolutives [5]. Le CA125 est le marqueur des cancers ovariens d’origine épithéliale [6]. Néanmoins, son taux peut être élevé dans plusieurs pathologies bénignes gynécologiques (endométriose, fibromes utérins, processus inflammatoires pelviennes), extra-gynécologiques (péritonite, pancréatite, hépatites, syndrome néphrotique, tuberculose péritonéale) ainsi que dans les cancers non gynécologiques avec métastases péritonéales. En cas de tuberculose péritonéale, des valeurs très élevées (>1000U/ml) peuvent se voir [7]. Dans l’étude de Koc et al, 90.1% des patientes atteintes de tuberculose péritonéale avaient un taux plasmatique du CA-125 élevé, et la valeur moyenne était 565 U/ml [8]. Par conséquent, le CA125 n’a pas de place dans le diagnostic différentiel entre cancer ovarien et tuberculose péritonéale. En revanche, Simsek et al. rapportent que la diminution du taux de CA125 est corrélée à la réponse au traitement antituberculeux et l’indiquent comme marqueur de surveillance sous traitement antibacillaire [9]. Les autres perturbations biologiques ne sont pas spécifiques notamment: l’anémie, le syndrome inflammatoire, de même que l’intradermo-réaction à la tuberculine. Le diagnostic de certitude par l’analyse du liquide d’ascite ne se fait qu’après la mise en évidence du Mycobacterium Tuberculosis soit à l’examen direct soit après culture sur milieu de Lowenstein-Jensen [10].

Dans notre cas, l’examen anatomo-pathologique extemporané après biopsie des granulations blanchâtres était suffisant pour confirmer le diagnostic. La recherche de la mycobactérie par polymerase chain reaction (PCR) peut être utile pour le diagnostic avec une sensibilité de 75 à 80% et une spécificité de 85 à 95%, mais cette technique est souvent indisponible [11]. Comme dans le cas de notre patiente, l´exploration chirurgicale s´impose devant la suspicion d´une tumeur maligne de l’ovaire. La voie d´abord peut être soit une laparotomie ou une laparoscopie. Des biopsies transvaginales ou transabdominales échoguidées peuvent être proposées en cas d’une forte suspicion de tuberculose, limitant ainsi les complications postopératoires [12]. L’étude histologique des biopsies permet de redresser le diagnostic en montrant des granulomes gigantocellulaires avec nécrose caséeuse spécifique du bacille de Koch. Le traitement de la tuberculose pelvienne est essentiellement médical. Il repose sur l’administration quotidienne d’une quadrithérapie associant: Isoniazide, Rifampicine, Ethambutol et la Pyrazinamide pendant deux mois, puis un traitement d´entretien pendant quatre mois par une bithérapie quotidienne associant l’Isoniazide la Rifampicine [13]. Un geste chirurgical est surtout indiqué en cas de masse compressive ou fistulisée pour mettre à plat les cavités caséifiées. La prévention de la tuberculose péritonéale repose sur la vaccination au bacille de Calmette et Guérin (BCG), le dépistage des formes asymptomatique, la chimioprophylaxie des sujets contacts et l´amélioration de l´hygiène de vie des populations à risque.

## Conclusion

La tuberculose péritonéale doit être évoquée devant toute masse ovarienne associée à un épanchement péritonéal. La cytologie et la culture du liquide de ponction d’ascite peuvent résoudre le problème. Dans le cas contraire, une cœlioscopie voir une laparotomie du fait des adhérences avec biopsie est indiquée permettant de redresser le diagnostic et d’éviter une chirurgie d’exérèse non justifiée le plus souvent chez une femme en activité génitale.
